# Structure of the staphylococcal enterotoxin B vaccine candidate S19 showing eliminated superantigen activity

**DOI:** 10.1107/S2053230X17014844

**Published:** 2017-10-20

**Authors:** Woo Hyeon Jeong, Dong Hyun Song, Gyeung Haeng Hur, Seong Tae Jeong

**Affiliations:** a The 5th R&D Institute, Agency for Defense Development, Yuseong PO Box 35, Yuseong-gu, Daejeon 34188, Republic of Korea

**Keywords:** SEB, staphylococcal enterotoxin B, T-cell receptor beta chain, recombinant protein, vaccines, bacterial superantigens

## Abstract

Four mutations (N23A, Y90A, R110A and F177A) were introduced into S19, a vaccine candidate for staphylococcal enterotoxin B (SEB), resulting in a lower binding affinity towards the T-cell receptor beta chain (TCB) and reducing its superantigen activity. The structure of S19 was solved and was superposed on the native or complex structure of SEB. In the superposition model, mutations that were introduced seemed to reduce the number of hydrogen bonds at the SEB–TCB interface. S19 also displayed an unexpected structural change around the flexible-loop region owing to the Y90A mutation. This local structural change provided evidence that the mutated form of S19 could have a lower affinity for major histocompatibility complex (MHC) class II than wild-type SEB.

## Abstract 2

Mutations introduced into S19, a recombinant staphylococcal enterotoxin B vaccine candidate, contribute to its reduced cytokine induction *in vivo* by removing hydrogen bonds and allowing interaction with the T-cell receptor beta chain and major histocompatibility complex class II.

## 1. Introduction   

Staphylococcal enterotoxin B (SEB) is one of seven toxins secreted by *Staphylococcus aureus*, and is classified as a bacterial superantigen (Balaban & Rasooly, 2000 ▸). SEB is one of the group II superantigens (Sundberg *et al.*, 2007 ▸), a group that includes SEC and SpeA. Superantigens in this group bind to T-cell receptors (TCRs) in a peptide-independent manner to generate an abrupt and strong immune response (Hewitt *et al.*, 1992 ▸; Fraser, 2011 ▸). This 28 kDa toxin consists of two domains, domain I (amino acids 30–120) and domain II (amino acids 127–239), which are composed of a β-barrel structure and antiparallel β-sheets, respectively. The structure of the MHC–SEB–TCB complex reveals the molecular mechanism by which it induces cytokine storms (Li *et al.*, 1998 ▸; Sundberg *et al.*, 2007 ▸). Previous studies have shown that SEB binds directly to MHC class II and TCB, thus hijacking the routine T-cell antigen-recognition process that involves antigen-presenting cells.

Previous research has investigated countermeasures against SEB, as it has caused numerous food-poisoning cases, especially in mess halls (Schmid *et al.*, 2009 ▸; Centers for Disease Control and Prevention, 2013 ▸). The United States Army Medical Institute of Infectious Diseases (USAMRIID) developed the recombinant vaccine STEBVax (Chen *et al.*, 2016 ▸) containing the mutations L45R, Y89A and Y94A, which reside at the MHC–SEB interface and prevent the production of a cytokine storm. Thus, vaccination with STEBVax may assure safety, and a Phase I clinical trial is ongoing.

We focused on interactions between the T-cell receptor beta chain (TCB) and SEB, which directly activate the immune system without involving MHC (Rödström *et al.*, 2014 ▸). We designed a vaccine candidate, S19, with reduced TCB–SEB interaction (Choi *et al.*, 2017 ▸) by introducing the following four mutations: N23A, Y90A, R110A and F177A. The candidate induces fewer cytokines upon *in vivo* administration, and in this study we provide the structure of S19 to explain the exact effect of the introduced mutations on its *in vivo* characteristics.

## 2. Materials and methods   

### 2.1. Macromolecule production   

The design of mutation sites in S19 was performed as described previously (Choi *et al.*, 2017 ▸). The cloning, expression and purification methods for S19 are described in Table 1 ▸. The target protein was lysed by sonication in buffer consisting of 20 m*M* Tris pH 8.0, 200 m*M* NaCl. The supernatant of the centrifuged lysate was loaded onto an Ni–NTA affinity chromatography column (GE Healthcare Life Science, Little Chalfont, England). After elution with buffer consisting of 20 m*M* Tris pH 8.0, 200 m*M* NaCl, 200 m*M* imidazole, the eluate was concentrated by ultrafiltration (Merck–Millipore, Billerica, Massachusetts, USA) and the macromolecule was finally purified to at least 95% purity by size-exclusion chromatography (SEC; Superdex 200, GE Healthcare Life Science) equilibrated with 20 m*M* Tris pH 8.0, 200 m*M* NaCl. The purity was determined using a Bioanalyzer (Agilent, Santa Clara, California, USA).

### 2.2. Crystallization   

Purified S19 was concentrated and crystallized using the following kits: The JCSG Core Suites I to IV (Qiagen, Hilden, Germany), The PACT Suite (Qiagen) and Structure Screen 1 + 2 (Molecular Dimensions, Suffolk, England). Details of the crystallization conditions are given in Table 2 ▸. The crystals were cryocooled in liquid nitrogen with 30% glycerol as a cryoprotectant.

### 2.3. Data collection and processing   

Cooled crystals were mounted and diffraction data were collected on beamline 7A at Pohang Accelerator Laboratory (PAL). Diffraction images were indexed and integrated using *HKL*-2000 (Otwinowski & Minor, 1997 ▸). Initial integration data were then scaled by *SCALEPACK*. *Phaser* (McCoy *et al.*, 2007 ▸) was used for molecular replacement prior to refinement. Detailed results and diffraction data are shown in Table 3 ▸. The data were initially indexed in a tetragonal lattice; however, they were re-indexed in an orthorhombic lattice since there was an NCS rotation parallel to the symmetry axis.

### 2.4. Structure solution and refinement   

The refinement process was performed using *PHENIX* (Adams *et al.*, 2010 ▸) and *Coot* (Emsley & Cowtan, 2004 ▸). The refinement statistics of S19 are given in Table 4 ▸.

### 2.5. Interface simulation between S19 and SEB with MHC   

The acquired structure of S19 was substituted with that of SEB from PDB entry 4c56 (Rödström *et al.*, 2014 ▸) using *LSQ Superpose* in *Coot*. This substituted model structure was validated with *ZDOCK* (Pierce *et al.*, 2014 ▸) by optimizing the docking interface between the components. The interface area of SEB–TCB/MHC was calculated by *PISA* (Krissinel & Henrick, 2007 ▸) and compared with the corresponding values from the substituted model.

## 3. Results and discussion   

### 3.1. Structural comparison between SEB and S19   

**Figure 1:**
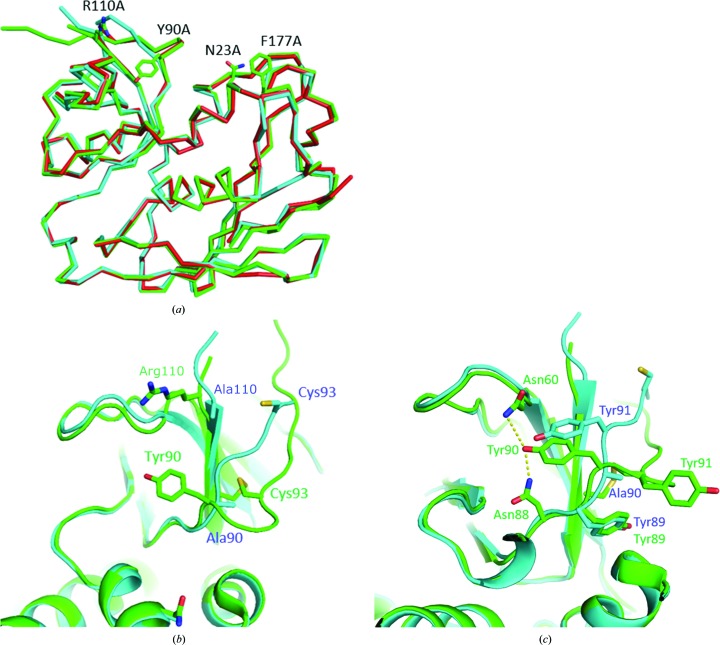
Overall structural comparison of SEB and S19. (*a*) LSQ superposition of the structures of MHC/TCB-bound SEB (green; PDB entry 4c56; Rödström *et al.*, 2014 ▸), unbound SEB (red; PDB entry 4rgm; Dutta *et al.*, 2015 ▸) and S19 (cyan). The original residues that are mutated in S19 are shown in stick form. (*b*) Structural changes in the flexible-loop region. The disulfide bridge between Cys93 and Cys113 is disrupted in the structure of S19. The C-terminal region of the loop region is not affected by the R110A mutation. (*c*) Side-chain structures related to the structural change. Tyr91 of S19 moves into the position occupied by Tyr90 in SEB, facilitated by hydrogen bonds to Asn60 and Asn88. Tyr89 does not move as a result of the Y90A mutation.

We compared the structures of S19 and SEB in the MHC–SEB–TCB complex (PDB entry 4c56). The overall structure of S19 is similar to that of SEB (Fig. 1 ▸ *a*), and there are no major structural changes in SEB upon complex formation. However, S19 exhibits some structural changes in its flexible-loop region (Tyr94–Thr113; Benedik *et al.*, 2014 ▸). Although the entire structure of the loop region has not been determined, owing to its outwards orientation and high flexibility, the N-terminal region of this loop is well stabilized by intramolecular hydrogen bonds and by a disulfide bridge between Cys93 and Cys113. The Y90A mutation was the sole reason for the observed structural change, as depicted in Fig. 1 ▸(*c*). Tyr91 of S19 moved into the original position of Tyr90, which had been stabilized by hydrogen bonds to Asn60 and Asn88. Tyr91 in S19 appeared to be stabilized by these interactions, supporting the conclusion that this movement was the major reason for the observed structural change. The structure and orientation of Asn88 and Tyr89 did not change between SEB and S19. The disulfide bridge in SEB was broken in S19 owing to the ∼8 Å translocation of Cys93 in S19. The purification and crystallization of S19 were performed under oxidative conditions to minimize reduction of the bridge. The R110A mutation was located on the C-terminus of the loop region but did not cause any noticeable surrounding changes (Fig. 1 ▸ *b*).

### 3.2. Simulated changes in binding interactions   

We confirmed that the Y90A mutation was the sole reason for the structural change in the loop structure of S19 and investigated the effect of this change on receptor binding. Since S19 exhibited a decrease in cytokine induction in a previous study, S19 has a lower affinity for either MHC class II or TCB (Rödström *et al.*, 2014 ▸).

**Figure 2:**
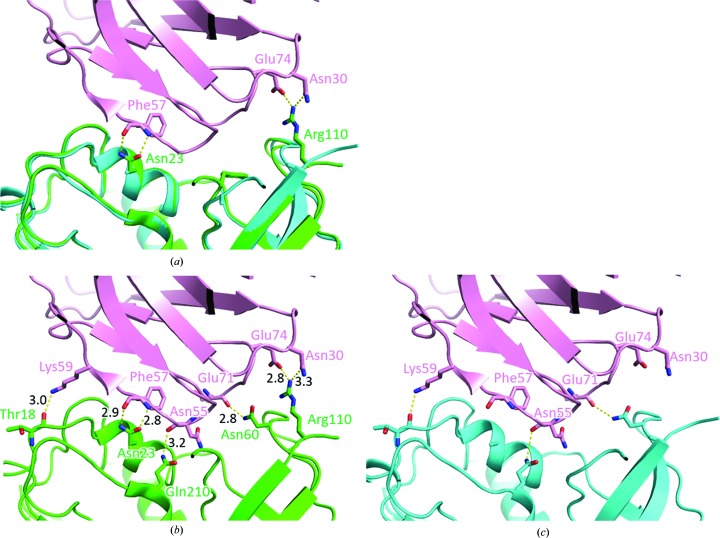
Modelled analysis of mutations at the TCB–SEB interface. (*a*) LSQ superposition of SEB and S19 showing the effects of mutation at the binding interface. Residues mutated in S19 and their hydrogen-bonding partners are shown in stick form. TCB, SEB and S19 are coloured pink, green and cyan, respectively. (*b*) Hydrogen bonds stabilizing the TCB–SEB binding interface. Participating residues are shown in stick form. (*c*) Estimated hydrogen bonds at the modelled TCB–S19 interface. The interface was modelled by LSQ superposition of S19 on PDB entry 4c56. Residues involved in the TCB–SEB binding interface are shown in stick form.

S19 was originally designed to make fewer molecular interactions with TCB than wild-type SEB, as a result of the removal of key interacting side chains. Since group II superantigens commonly target the Vβ domain of the T-cell receptor, we introduced N23A, R110A and F177A mutations (Kappler *et al.*, 1992 ▸; Fields *et al.*, 1996 ▸; Li *et al.*, 1998 ▸). Fig. 2 ▸(*a*) depicts the hydrogen bonds formed by the residues and the corresponding superposition of S19. The following residues in SEB form hydrogen bonds: Thr18, Asn23, Asn60, Arg110 and Gln210 (Fig. 2 ▸ *b*). The modelled interface between S19 and TCB (Fig. 2 ▸ *c*) predicted that more than half of the interactions mentioned would be diminished by the designed mutations. Thus, the superposition model showed a decreased affinity of S19 for TCB compared with wild-type SEB, which would explain the reduced cytokine induction of S19. The lengths of the hydrogen bonds in Fig. 2 ▸(*c*) are omitted owing to modelling limitations.

**Figure 3:**
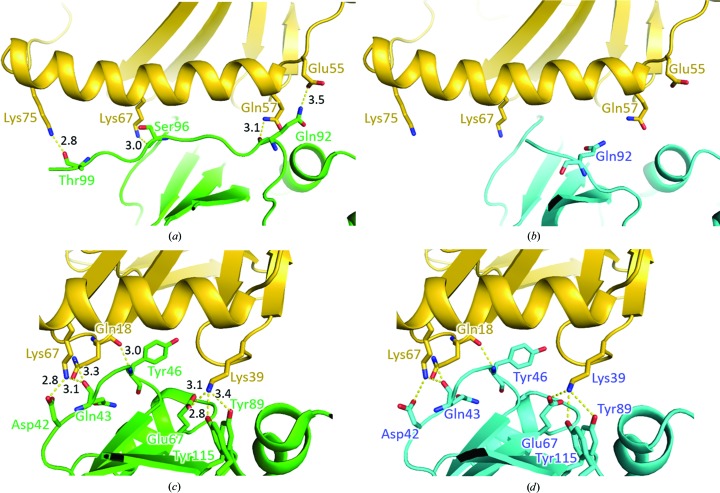
Modelled analysis of mutations at the MHC–SEB interface. (*a*) Hydrogen bonds between MHC and the flexible-loop region of SEB. Residues forming hydrogen bonds between MHC and SEB are shown in stick form. MHC, SEB and S19 are coloured yellow, green and cyan, respectively. (*b*) Modelled interface between MHC and S19. Residues shown in stick form in (*a*) are also shown in stick form here. (*c*) Hydrogen bonds between MHC and SEB, excluding the flexible-loop region. Residues forming hydrogen bonds between MHC and SEB are shown in stick form. (*d*) Modelled interface between MHC and S19 corresponding to (*c*). Residues shown in stick form in (*c*) are also shown in stick form here. The lengths of hydrogen bonds are omitted as they are modelled by superposition.

A previous study of the interactions between SEB and MHC class II showed that the loop region participates in intermolecular hydrogen bonds and hydrophobic interactions (Jardetzky *et al.*, 1994 ▸; Yanaka *et al.*, 2010 ▸). Thus, we categorized the interface between MHC class II and SEB into two parts, one of which contained the loop region. As the loop structure in S19 is significantly distorted, the intermolecular interactions mediated by Gln92 were not modelled in S19 (Figs. 3 ▸ *a* and 3 ▸ *b*). However, owing to its increased flexibility and the absence of a binding partner, we could not determine the structure of the loop region from Ser96 to Thr99 in S19. Additional studies are needed to determine the structure of the complex of S19 and MHC.

At its interface without the loop region, S19 has almost the same backbone structure as SEB. There are very few differences in the structures of SEB and S19, and the hydrogen bonds mediating the interface appear to be conserved after the mutations (Figs. 3 ▸ *c* and 3 ▸ *d*). Since one mutation accidentally changed the loop structure that participates in the MHC–SEB interaction, the binding affinity may be affected.

The changes in the interfaces observed in the TCB–SEB–MHC complex on substituting SEB by S19 were further measured *in silico* using *PISA*. The interface areas between TCB and SEB and between MHC and SEB were decreased 27 or 14% by the mutations, respectively; thus, the simulation reflected the loss of binding residues. The number of simulated hydrogen bonds also decreased from 10/10 to 8/5, respectively.

In this study, we measured the molecular structure of S19 to explain how the designed mutations contribute to the reduced *in vivo* cytokine induction of S19. S19 maintained a similar overall structure to SEB, which is required for its antigenicity as a vaccine candidate. However, the Y90A mutation caused a shift in the position of Tyr91, consequently inducing a structural change in the flexible-loop structure. A broken disulfide bridge in S19 contributed to this structural change but was stabilized by hydrogen bonds generated by Tyr91, a role that is played by Tyr90 in SEB. S19 was originally designed to have a lower binding affinity for TCB, but structural determination revealed reduced interactions with both binding partners, TCB and MHC. Although the LSQ superposition model provided an empirical clue to the loss of superantigen properties in S19, the actual change of affinity towards MHC or TCB should be determined to confirm the exact role of the mutations.

## Supplementary Material

PDB reference: staphylococcal enterotoxin B mutant S19, 5xz0

## Figures and Tables

**Table 1:** Macromolecule-production information

Source organism	*S. aureus*
DNA source	Synthetic
Forward primer	TCACTACCATATGAAAGCCAGCCTGATCCGAAACCG
Reverse primer	ACTACGCGGCCGCTCATTTTTTGGTGGTCAGATACACCTC
Cloning vector	pET-28a
Expression vector	pET-28a
Expression host	*Escherichia coli*
Purification method	Ni–NTA affinity chromatography and SEC (Superdex 200)
Complete amino-acid sequence of the construct produced	SQPDPKPDELHKSSKFTGLMEAMKVLYDDNHVSAINVKSIDQFLYFDLIYSIKDTKLGNYDNVRVEFKNKDLADKYKDKYVDVFGANYAYQCYFSKKTNDINSHQTDKAKTCMYGGVTEHNGNQLDKYRSITVRVFEDGKNLLSFDVQTNKKKVTAQELDYLTRHYLVKNKKLYEANNSPYETGYIKFIENENSFWYDMMPAPGDKFDQSKYLMMYNDNKMVDSKDVKIEVYLTT

**Table 2:** Crystallization of S19

Method	Sitting-drop vapour diffusion
Plate type	MRC Crystallization Plate
Temperature (K)	277 or 298
Protein concentration (mg ml^−1^)	10
Buffer composition of protein solution	20 m*M* Tris–HCl pH 8.0, 200 m*M* NaCl
Composition of reservoir solution	0.2 *M* KF, 20%(*w*/*v*) PEG 3350
Volume and ratio of drop	300 nl, 1:1
Volume of reservoir (µl)	50

**Table 3:** Data collection and processing Values in parentheses are for the outer shell.

Diffraction source	Beamline 7A, PAL
Wavelength (Å)	0.97933
Temperature (K)	100
Detector	ADSC Quantum 270
Crystal-to-detector distance (mm)	300
Rotation range per image (°)	1
Total rotation range (°)	180
Exposure time per image (s)	0.5
Space group	*C*222
*a*, *b*, *c* (Å)	174.6, 174.6, 48.8
α, β, γ (°)	90.0, 90.0, 90.0
Mosaicity (°)	0.685
Resolution range (Å)	50–3.00
Total No. of reflections	94032
No. of unique reflections	15415
Completeness (%)	99.8 (99.9)
Multiplicity	6.1
〈*I*/σ(*I*)〉	16.5 (2.97)
*R*_r.i.m._	0.053 (0.207)
Overall *B* factor from Wilson plot (Å^2^)	43.9

**Table 4:** Structure solution and refinement Values in parentheses are for the outer shell.

Resolution range (Å)	50–3.00
Completeness (%)	99.4
σ Cutoff	1.4
No. of reflections, working set	13867
No. of reflections, test set	1548
Final *R*_cryst_	0.204
Final *R*_free_	0.243
Cruickshank DPI	0.256
No. of non-H atoms	
Protein	3668
Ion	0
Ligand	0
Water	34
Total	3702
R.m.s. deviations	
Bonds (Å)	0.003
Angles (°)	0.500
Average *B* factor (Å^2^)	47.2
Ramachandran plot	
Most favoured (%)	95.18
Allowed (%)	4.82
